# Simultaneous Detection of Viability and Concentration of Microalgae Cells Based on Chlorophyll Fluorescence and Bright Field Dual Imaging

**DOI:** 10.3390/mi12080896

**Published:** 2021-07-29

**Authors:** Yanjuan Wang, Junsheng Wang, Tianqi Wang, Chengxiao Wang

**Affiliations:** 1 Software Institute, Dalian Jiaotong University, Dalian 116028, China; wangyanjuan@djtu.edu.cn (Y.W.); wtq@dlmu.edu.cn (T.W.); aad1957049044@163.com (C.W.); 2 Center of Microfluidic Optoelectronic Sensing, Dalian Maritime University, Dalian 116026, China; 3 College of Information Science and Technology, Dalian Maritime University, Dalian 116026, China

**Keywords:** chlorophyll fluorescence, bright field, ship ballast water, microalgae, detection

## Abstract

Ship ballast water contains high concentration of plankton, bacteria, and other microorganisms. If the huge amount of ballast water is discharged without being inactivated, it will definitely spell disaster to the marine environment. Microalgae is the most common species exiting in ballast water, so the detection of the concentration and viability of microalgae is a very important issue. The traditional methods of detecting microalgae in ballast water were costly and need the help of bulky equipment. Herein, a novel method based on microalgae cell intracellular chlorophyll fluorescence (CF) imaging combines with cell bright field (BF) microscopy was proposed. The geometric features of microalgae cells were obtained by BF image, and the cell viability was obtained by CF image. The two images were fused through the classic image registration algorithm to achieve simultaneous detection of the viability and concentration of microalgae cells. Furthermore, a low-cost, miniaturized CF/BF microscopy imaging prototype system based on the above principles was designed. In order to verify the effectiveness of the proposed method, four typical microalgae in ballast water (*Platymonas*, *Pyramimonas* sp., *Chrysophyta*, and *Prorocentrum lima*) were selected as the samples. The experimental results show that the self-developed prototype can quickly and accurately determine the concentration and the viability of microalgae cells in ship ballast water based on the dual images of BF and CF, and the detection accuracy is equivalent to that of commercial microscope. It was the first time to simultaneously detect the viability and concentration of microalgae cells in ship ballast water using the method that combining the fluorescence and bright field images; moreover, a miniaturized microscopic imaging prototype was developed. Those findings expected to contribute to the microalgae detection and ship ballast water management.

## 1. Introduction

Ship ballast water is the sea water loaded in the cabin to control the draught of the hull and improve the stability and maneuverability of the ship. When ships berth at the wharf, the ballast water must be loaded or discharged to ensure smooth sailing [1,2]. It is estimated that more than 10 billion tons of ballast water was transferred by ships in the world every year. Ship ballast water contains a lot of bacteria, viruses, and plankton. If the huge number of organisms in the ballast water directly discharged into the destination sea without inactivated, it will inevitably cause the invasion of alien species and spell disastrous consequences for the ecological environment [3,4,5,6,7]. Therefore, ship ballast water must be inactivated before being discharged [8,9,10].

Recognized the seriousness of the problem, International Maritime Organization (IMO) convened the international convention on the control and management of ship ballast water and sediment in February 2004. The convention stipulates that the ballast water that will be discharged by ship should meet desired standards. This article will focus on the application of Standard D-2 performance. The number of alive organisms between 10 μm and 50 μm per milliliter of ballast water must be less than 10 [11,12,13]. In September 2017, the ballast water management convention has entered into force. In order to meet the discharge standards, ships need to install approved ballast water management systems and the corresponding detection of ship ballast water must be carried out in accordance with the provisions of the IMO [14]. Microalgae is the typical organisms in ship ballast water, so the detection of the concentration and viability of microalgae is a major issue related to ship ballast water management and marine environmental protection.

The traditional detection methods of microalgae viability and concentration include microscopy, fluorescence staining, Coulter counter, flow cytometry, and so on. For microscopic detection, the microalgae cells must be stained with neutral red or other stains. For the dead cells, the rupture of the cytomembrane causes the stains enter and stain the cells, and then can be observed and counted manually under the microscope [15,16]. This method is simple and reliable, and can realize the simultaneously detection of the viability and quantity of microalgae cells, which is the classical and benchmark method at present. However, this method requires professional operators to carry out in the laboratory, which is time-consuming and laborious. Moreover, during the transportation process, samples may be damaged by temperature, light, etc. Fluorescent labeling is another commonly used method [17], in that, the dead or alive cells will be labeled with specific fluorescent dye, and the viability of the cells can be observed and judged manually under a fluorescence microscope [18,19]. The Coulter counter detects the quantity and size of particles by detecting the resistance changes between the two electrodes [20]. It is fast and highly automated, but it cannot determine whether the substance being detected is phytoplankton or particles, nor can it detect its viability. Moreover, the commercial Coulter counter is bulky and expensive, it must be performed in a laboratory and cannot be used for on-site detection [21]. Flow cytometry is another commonly used detection method in the laboratory, by means of fluorescence staining, it can accurately detect and analyze the viability and quantity of cells [22,23]. However, this method is also expensive, bulky, complicated to operate, high maintenance cost, and difficult to carry out in resource-poor areas [24].

To sum up, the traditional methods are costly, time-consuming and need to be carried out in the laboratory with the help of bulky equipment, which cannot realize the on-site detection. Benefit from the availability of low-cost, compact and high-performance image sensors, numerous compact lens-free imaging systems have been reported, and tremendous amount of advancements have been made in the miniaturized microscopic imaging system. For example, Zhang et al. [25] developed a miniature microscope from off-the-shelf components and webcam, which was able to chronologically monitor cell migration and analyze beating of microfluidic liver and cardiac bioreactors in real time. Kim et al. [26] provides a portable fluorescence device for the identification of fluorescently-labeled somatic cells, and has been successfully applied in the detecting and counting of somatic cells in milk. Dai et al. [27] demonstrated a handheld smartphone fluorescence microscope that can be attached to the smartphone camera for both bright-field and fluorescence imaging at cellular-scale resolutions. Liu et al. [28] proposed a catadioptric microscope objective lens that features an integrated MEMS device for performing biaxial scanning, axial focus adjustment, and control of spherical aberration. In addition, various advances have been made in improving the imaging resolution [29,30,31], enhancing the sensitivity of the sensors [32,33]. However, little research has been done on the miniaturized micro equipment for the detection of microalgae cells in ship ballast water, and its viability.

A tremendous amount of ballast water needs to be detected every year. In order not to delay the sailing time of the ships, the ship ballast water detection needs to be carried out quickly on site. Microalgae is the most common organisms in ship ballast water, so how to quickly and accurately detect microalgae cells in ballast water is an important issue for ship ballast water management. However, there are few reports on miniaturized equipment or methods for rapid detection of the viability and concentration of microalgae cells. Therefore, there is an urgent need to develop a low-cost, portable, easy-to-operate method that can rapidly detect the viability and concentration of microalgae cells in ship ballast water.

It is known that the photosynthetic capacity of plant cells reflects its vitality. Chlorophyll is the key biomolecule for plants to absorb light energy and plays an important role in algae photosynthesis. The chlorophyll spectrum in plant cells has the absorption peak and emission peak [34]. When being irradiated by excitation light with a wavelength equal to the absorption peak of the chlorophyll spectrum, the chlorophyll in the cell can absorb the energy of the monochromatic light and emitting an optical signal with a wavelength of the emission peak, that is, the CF signal. Studies have shown that the signal intensity of the CF is directly proportional to the amount of chlorophyll in the cells, thus, which can be used to evaluate the photosynthetic capacity and the viability of cells [35].

Inspired by these studies, we herein proposed a novel method to detect the viability and concentration of microalgae cells simultaneously by combining the CF and BF images. The fluorescence intensity of CF image of microalgae cells was used to characterize the viability of cells; the geometric characteristics and the concentration of cells were characterized by the BF image. Through the fusion of CF and BF dual images, the viability and concentration of microalgae cells can be detected simultaneously. Based on the above principles, we developed a miniaturized and portable BF/CF dual microscopic imaging system using low-cost CMOS components [36,37]. The micro resolution of the system is 2.19 μm, the working distance is 2.4 mm, and the magnification is equivalent to a 10× objective lens of commercial microscope. It costs less than $150 and weighs just 221.8 grams (See the supplementary materials for the cost list of this system). The system has two imaging modes: bright field and fluorescence. Under the fluorescence imaging mode, the CF image of the microalgae cells can be obtained, which reflects the cytoactive and can be used to evaluate the inactivation effect of the ship ballast water. Under the bright-field mode, the BF image of the microalgae cells can be obtained. Through the self-developed counting software, the quantity of the cells can be automatically obtained, thereby the concentration of cells can be calculated. By fusing the CF and BF dual images, the viability and concentration of the microalgae cells in ballast water can be obtained simultaneously. In our research, four typical microalgae in ballast water (*Platymonas*, *Pyramimonas* sp., *Chrysophyta*, and *Prorocentrum lima*) were selected as the samples to verify the effectiveness of the proposed method and the prototype system. In this paper, the miniaturized system was compared with the commercial microscope in several aspects, such as bright field resolution, fluorescence imaging ability, cell concentration detection and cell viability estimation. The experimental results show that the system is functionally comparable to commercial microscopes, but it is cheaper, smaller and more portable. In addition, the system adopts non-contact and non-invasive detection method, which is harmless to cells.

It is the first time to simultaneously detect the concentration and viability of the microalgae cells in ballast water based on the fusion of CF and BF dual images of microalgae cells, using a self-developed low-cost, miniaturized microscopic imaging equipment. This article provides a low-cost, high-efficiency, and on-site solution for the detection of microalgae cells in ship ballast water, which may have great significance for the marine environment protection and the prevention of red tide disaster. In addition, with the help of micro/nano-fabrication techniques and integrated micro/nano-optics [38,39,40], using super-resolution imaging technology [41,42,43], more novel and potential devices can be realized. The system can be more miniaturized, intelligent, high-precision, and the application range of the system will be wider.

## 2. Materials and Methods

### 2.1. Principle and System Design

In our research, the viability and concentration of microalgae in ship ballast water were detected simultaneously based on the CF and BF dual images. A commercial CMOS imaging module and its optical lens were used to construct a dual imaging micro-system. From the optical imaging principle of the lens, that the distance between the lens and the sensors determines the magnification. The further the distance, greater the magnification. In order to achieve the amplification, the lens in the CMOS module was inverted here [25]. The optical path structure used in this system is a transmissive structure.

**Figure 1:**
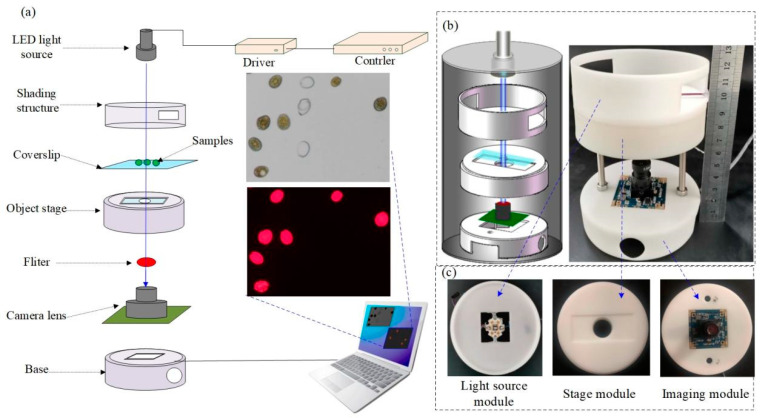
(**a**) Structure of the dual imaging microscopy system. (**b**) System design and the photograph. (**c**) Photographs of the light source module, the stage module and the imaging module.

The structure of the designed BF/CF dual imaging microscopy system is shown in Figure 1a. The system is composed of four main modules: light source module, stage module, imaging module, and image processing module. The light source module is composed of fixed structure and a hi-light LED (LZ1-00DB00 LED Engin, Inc. San Jose, CA USA). The system has two imaging modes: bright field and fluorescence. In the fluorescence imaging mode, the wavelength of excitation light used was 488 nm, because the absorption peak of chlorophyll spectrum in microalgae cells is 488 nm and the emission peak is 685 nm. That is, the microalgae cells can emit CF signals of 685 nm under the excitation of 488 nm monochromatic light [44]. The CF signal was received by the CMOS imaging module. If the other wavelengths of fluorescence signals need to be observed, simply adjust the excitation wavelength and change the filter, so, our system can be extended to other applications. The role of the stage module is to load cells, and provides the required optical path for the system. A 24 × 50 mm^2^ rectangular groove was designed on the stage for loading the coverslip. A hole with a diameter of 2 cm was designed in the center of the groove for light transmission. The imaging module is composed of CMOS sensor, filter and fixed structure. The CMOS sensor was disassembled from a HD USB camera, the minimum illumination of the camera was 0.01 lx, and the photosensitive area was 1/2.9” (diagonal length 6.23 mm). The CMOS sensor (Sony IMX322) has 1920 × 1080 pixels, and the pixel pitches is 2.8 × 2.8 μm^2^. The collected images were transferred to the computer through a USB interface for subsequent processing. Under fluorescence imaging mode, a 685 nm filter (EX685/50 M, Chroma ATE Inc., Novi, MI, USA) was placed above the CMOS sensor to receive the CF signals and minimize the interference of stray light. The image processing module consists of a computer and a self-developed cell counting system. Figure 1b shows the system design and the photograph. The main structure of the system was drawn with SolidWorks software, and then printed by 3D printing technology. The entire system requires close integration of all parts, good airtightness, to ensure that it will not be interfered by external light, and to minimize the light loss. The microscopic imaging system is cylindrical, with a base diameter of 9 cm, a height of 12.5 cm, and a weight of 221.8 g. which is highly portable. Figure 1c shows the photographs of the light source module, the stage module and the imaging module.

### 2.2. Viability Detection of Microalgae Cells

As mentioned earlier, the chlorophyll in microalgae cells will emit CF signal with the wavelength of 685 nm under the excitation of light with the central wavelength of 488 nm. When using sodium hypochlorite (NaClO) solution to inactivate microalgae cells, NaClO would hydrolyze and infiltrate into the microalgae, oxidize and destroy the DNA, RNA, and metabolic enzymes of the cells, and destroy chlorophyll, thus leading to cell death. For the dead cells, it will not absorb light energy and emit CF signals. Moreover, the viability of microalgae is proportional to the intensity of the CF signal, so we can analyze the viability of microalgae by measuring the intensity of fluorescence signal. This system obtains the CF image of microalgae cells under the fluorescence imaging mode, and measure the average fluorescence intensity of the images using ImageJ software (National Institutes of Health, Bethesda, MD, USA).

### 2.3. Concentration Detection

In order to detect the concentration of microalgae cells in the ballast water, a hemocytometer was used to load the samples instead of the coverslip. The BF images of microalgae cells were obtained under the bright-field imaging mode of this system, then the number of cells and the concentration of samples were obtained by the self-developed counting software.

### 2.4. Sample Preparation

The samples used here were *Platymonas* (10–15 μm in diameter), *Pyramimonas* sp. (10–15 μm in diameter), *Chrysophyta* (4–6 μm in diameter), *Prorocentrum lima* (20–40 μm in diameter), all purchased from Liaoning Ocean and Fisheries Science Research Institute (Dalian, China). Each microalgae species was cultured individually in a conical flask containing enriched seawater medium. Then, 15 mL of the four microalgae species were taken and centrifuged for 10 mins (the speed was 8000 rpm, the temperature was 20 °C). Then, pour out the supernatant and put the algae cells into a 1.5 mL tube, add the prepared phosphate buffer saline (PBS) solution, then shake evenly for use.

For dead microalgae cells, the inactivation method was chlorination. For different microalgae cells, different concentrations of NaClO solution were added, and the dead cells can be obtained after evenly mixed and standing for 20 min.

### 2.5. Experimental Setup

Before the experiment, the coverslip should be cleaned with absolute alcohol. In order to avoid the influence of residual alcohol, the coverslip must be flushed with the prepared PBS solution for 1 min, then dried with nitrogen, and placed it in the groove of the stage. The prepared microalgae cell solution was taken and shook evenly. Then, a pipette was used to drop 10 µL microalgae solution on the coverslip and covered it with another cleaned coverslip, and adjust the focal length of the system. When obtaining the BF images, the ambient light was used as the light source. For the CF images, the HD LED light source with the central wavelength of 488 nm was used. In order to reduce the interference of stray light, a 685 nm filter (EX685/50) was added to receive the CF signal of the microalgae. When obtaining the CF image of cells, the experiment should be carried out in a dark environment.

### 2.6. Cell Image Acquisition and Analysis

In order to automatically count the number of the cells in images, we developed a cell counting system. It includes the following steps: image preprocessing (denoising, adjusting the gray level range), adaptive setting of image threshold, image binarization, image segmentation, morphological processing, calculation of isolated objects, etc. Through this self-developed counting software, the concentration of microalgae cells in ballast water can be obtained automatically and quickly.

### 2.7. Image Registration and Fusion

In order to achieve simultaneous detection of the concentration and the viability of the sample, image registration and fusion are required. In this paper, an image registration algorithm based on grayscale information was adopted. The gray relationship of two images was used for registration, and the weighted fusion algorithm was used for image fusion. Thought the two classic image registration algorithms, the fusion of the BF and CF images can be achieved (see the supplementary materials for the details).

## 3. Results and Discussion

### 3.1. Resolution Analysis of Miniaturized Dual Imaging Microscopy System

**Figure 2:**
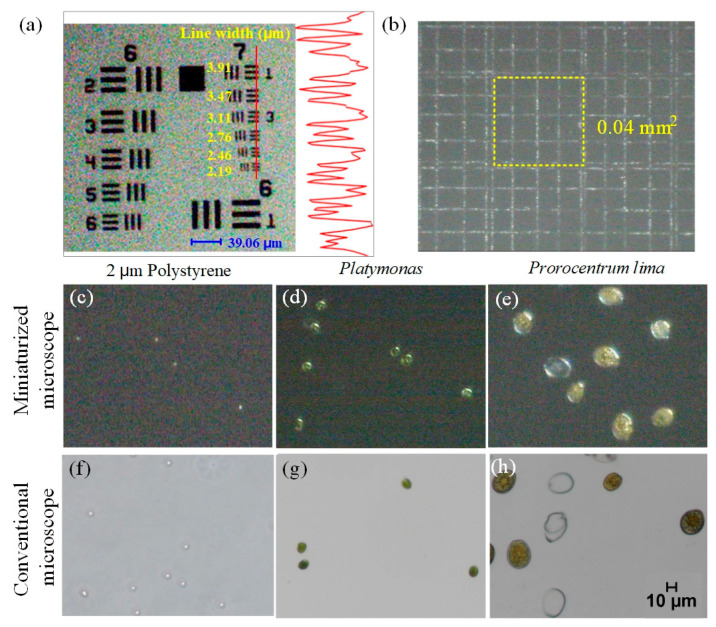
(**a**) Positive resolution test target, the red line is a transect and that the adjacent plot shows gray scale modulation across the line pairs on the resolution test chart. (**b**) Field of view of the miniaturized microscopic system. (**c**–**e**) The BF images of the polystyrene particles (with the diameter of 2 µm) and two common microalgae cells (*Platymonas* and *Prorocentrum lima*) captured by the miniaturized microscope. (**f**–**h**) shows the comparison of those corresponding samples captured by a commercial microscope.

In order to test the microscopic resolution of the dual imaging microscope system, a positive resolution test target (1951 USAF Thorlabs) was used here. As shown in Figure 2a, the system can clearly obtain the image of the sixth element in the seventh group on the test target. Then the ImageJ software was used to display the line scan of the BF image. The red line is a transect and that the adjacent plot shows gray scale modulation across the line pairs on the resolution test chart. It can be seen, that the miniaturized system was able to resolve lines as closely spaced as 2.19 μm with clear peak separation. Such a high resolution is sufficient for the ship ballast water and most cell detection applications.

In order to measure the field of view of the miniaturized system, we imaged the hemocytometer and calculated the frame size based on the grid. The result was shown in Figure 2b. The area of a smallest cell on the hemocytometer is 0.0025 mm^2^. The yellow square in Figure 2b contains 16 small cells, so its area is 0.04 mm^2^. By calculating the ratio of the frame size of the image to the yellow square, the field of view area can be calculated. After calculation, the field of view of the system is 0.397 mm^2^. Figure 2c–e shows the BF images of the polystyrene particles (with the diameter of 2 µm) and two common microalgae cells (*Platymonas* and *Prorocentrum lima*) captured by the miniaturization system under bright-field mode. Figure 2f–h shows the comparison of the corresponding samples captured by a commercial microscope (Nikon Eclipse Ti2-E, Nikon corporation Tokyo, Japen) under 10× objective lens. As shown in those figures, the system can image both 2 µm polystyrene particles and algae cells clearly, and the image quality is comparable to that of a commercial microscope under 10× objective lens.

### 3.2. Fluorescence Analysis of Miniaturized Dual Imaging Microscopy System

**Figure 3:**
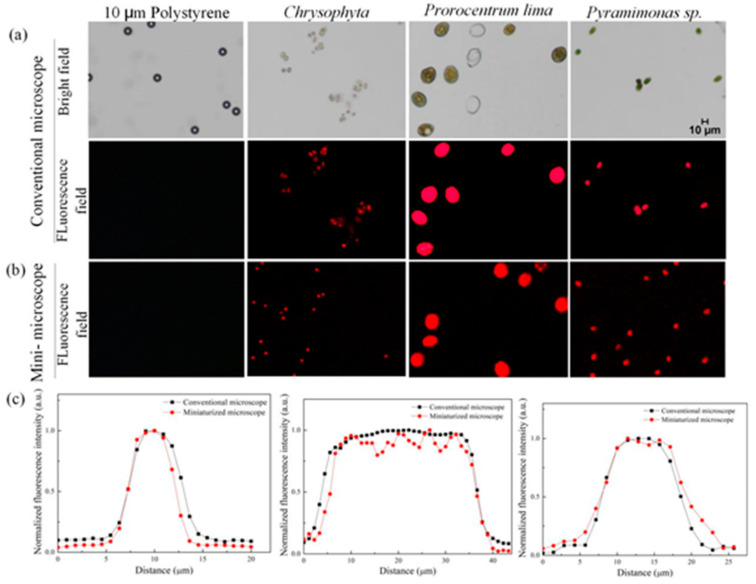
(**a**) Bright field (BF) and chlorophyll fluorescence (CF) images of the four samples captured by commercial microscope (10 μm particles, *Chrysophyta*, *Prorocentrum lima*, and *Pyramimonas* sp.). (**b**) CF images of the four samples captured by the miniaturized system. (**c**) Fluorescence intensity distribution curves of the *Chrysophyta*, *Prorocentrum lima*, and *Pyramimonas* sp.

As shown in Figure 3, polystyrene particles (10 μm diameter), *Chrysophyta*, *Prorocentrum lima*, and *Pyramimonas* sp. were used as the samples to verify the fluorescence ability of this micro-system. The purpose of introducing the ordinary polystyrene particles was to provide a control group. Because polystyrene particles have no fluorescence properties, the signals of polystyrene particles cannot be captured under the fluorescence mode, while the microalgae cells have the characteristics of chlorophyll fluorescence and can stimulate CF signal under the excited by light with a wavelength of 488 nm. So, CF images of microalgae cells can be captured. Figure 3a shows the BF and CF images of the four samples captured by a commercial microscope, and Figure 3b shows the CF images of the four samples captured by the miniaturized system. It can be seen that neither the miniaturized system nor the commercial microscope can capture the fluorescence signal of the polystyrene particles, but both of them can obtain the CF images of the microalgae cells. As can be seen from the figures, the quality of the CF images obtained by the miniaturized system is comparable to that of the commercial microscope.

In order to further verify the fluorescence capability of our miniaturized system, ImageJ software was used to perform single-cell line scans on the CF images of the three kinds of microalgae cells. The fluorescence intensity distribution curves were shown in Figure 3c. From left to right, there were *Chrysophyta*, *Prorocentrum lima*, and *Pyramimonas* sp. It can be seen from the figures that the fluorescence intensity distribution curves of the three kinds of cells captured by the miniaturized system were consistent with that of the commercial microscopes.

Furthermore, the full-width at half maximum (FWHM) of the fluorescence intensity distribution curve of the three kinds of microalgae cells were calculated, and the results were shown in Table 1.

From Table 1, the difference between the FWHM of the fluorescence intensity distribution curve obtained by this system and that of the commercial microscope is within 5%. The above results further demonstrated that the miniaturized system designed here has the comparable fluorescence imaging capability with the commercial microscope.

### 3.3. Concentration Detection of Microalgae Cell in Ballast Water

**Figure 4:**
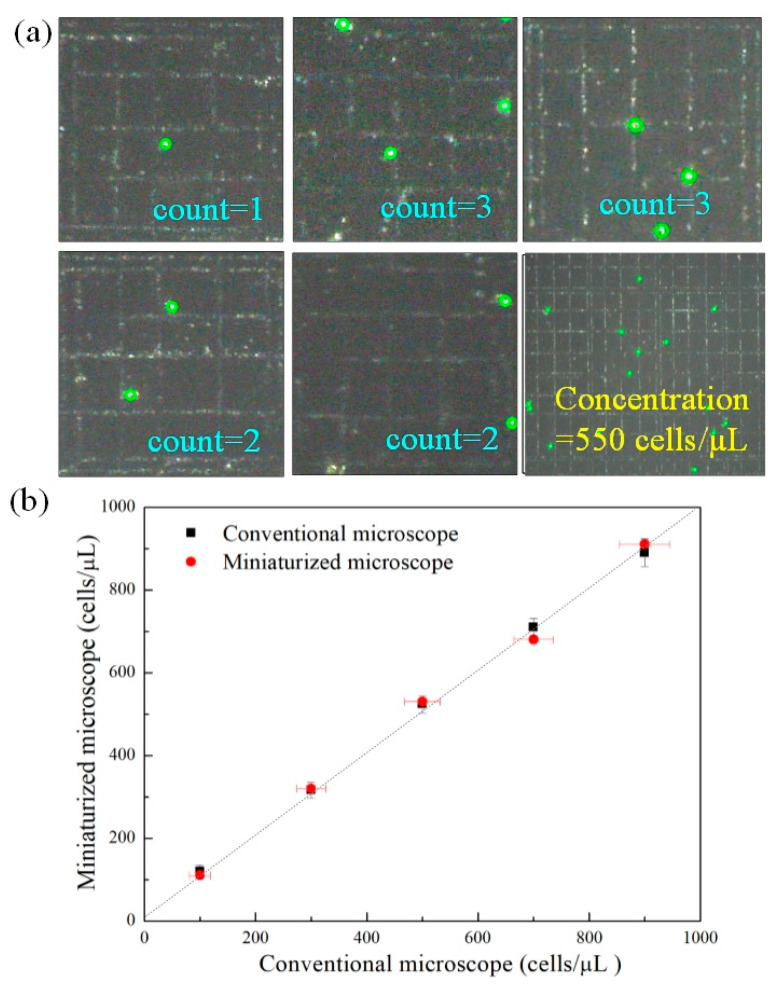
(**a**) BF images of the five counting units of the hemocytometer (upper left, upper right, middle, lower left and lower right). (**b**) Concentration detection by the miniaturized system and a commercial microscope.

Concentration detection of microalgae is one of the key points of ship ballast water management. Microscopy is the standard method for detecting cell concentration, but it is time-consuming and laborious. In order to detect the concentration of microalgae in ship ballast water quickly and accurately, an automatic concentration detection method was proposed here. A hemocytometer was used instead of the coverslip to load microalgae samples, and the BF images of five counting positions on the hemocytometer were taken respectively. A self-developed cell counting system was used to automatically count the number of the cells and calculate the concentration of the samples. Figure 4a shows the BF images of the five counting units of the hemocytometer, there were upper left, upper right, middle, lower left, and lower right, respectively. In order to evaluate the accuracy of the cell concentration detection of this system, the following experiments were carried out. Five different cell concentrations (100, 300, 500, 700, and 900 cells/µL, *Platymonas* cells) were prepared, and the same samples were detected with the miniaturized system and a commercial microscopic respectively. The result was shown in Figure 4b (n = 3, n is the number of replicates). It can be seen, that the accuracy of the cell concentration detection by this system is comparable to that of the microscope. The experiments show that compared with the microscope, the detection error of the system is within 3.5% (see the Supplementary Materials for the details). The above results show that the system can provide a simple, rapid and reliable solution for the detection of microalgae cell concentration in ship ballast water.

### 3.4. Viability Estimation of Microalgae Cells

In order to comply with the D-2 standard, ship ballast water must be inactivated before discharged. Therefore, how to estimate the viability of microalgae cells quickly and accurately is an important issue of the ship ballast water management. Herein, the optical-induced chlorophyll fluorescence principle was used to evaluate the viability of microalgae cells. As mentioned above, the chlorophyll of microalgae cells will emit the CF signals being irradiated by the light of specific wavelength, and the viability of the cells is proportional to the intensity of the CF signal. The NaClO inactivation method is a commonly used method for inactivating microorganisms in ship ballast water, it is low cost, high efficiency, and easy to operate. NaClO solution will penetrate into cells, oxidize and destroy the cells, causing cell death. 

**Figure 5:**
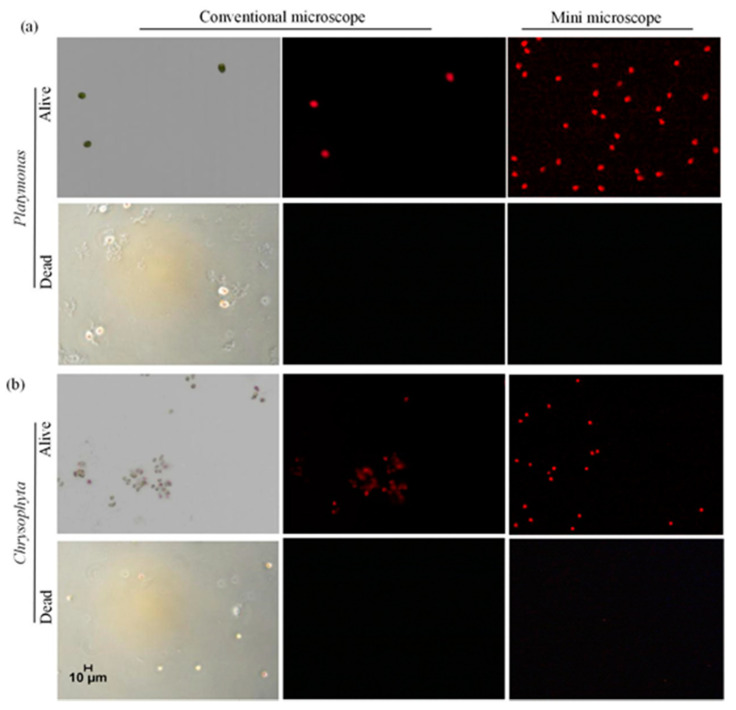
BF and CF images of the living and dead cells captured by commercial microscope and the miniaturized system. (**a**) *Platymonas* cells. (**b**) *Chrysophyta* cells.

First, we used the living and dead (inactivated by NaClO solution) *Platymonas* and *Chrysophyta* cells as the samples, and the BF and CF images of both cells were captured by the commercial microscope and our system. The experimental results were shown in Figure 5. Figure 5a was a group of images of *Platymonas* cells, and Figure 5b is that of *Chrysophyta* cells. As can be seen from the figures, under the bright field mode, for the dead cells, due to the strong oxidation of NaClO, both kinds of algal cells changed in color, but the shape and size of the cells remained, and the contour of the dead cells remained intact. Under the fluorescence mode, for living *Platymonas* cells and *Chrysophyta* cells, both commercial microscope and the system can capture strong CF signals. For dead cells, neither of the two methods can capture the CF signal of the cells, because the chlorophyll in the cells has been destroyed by NaClO. The above results demonstrated that our system can detect whether a microalgae cell is alive or dead based on its CF signal.

**Figure 6:**
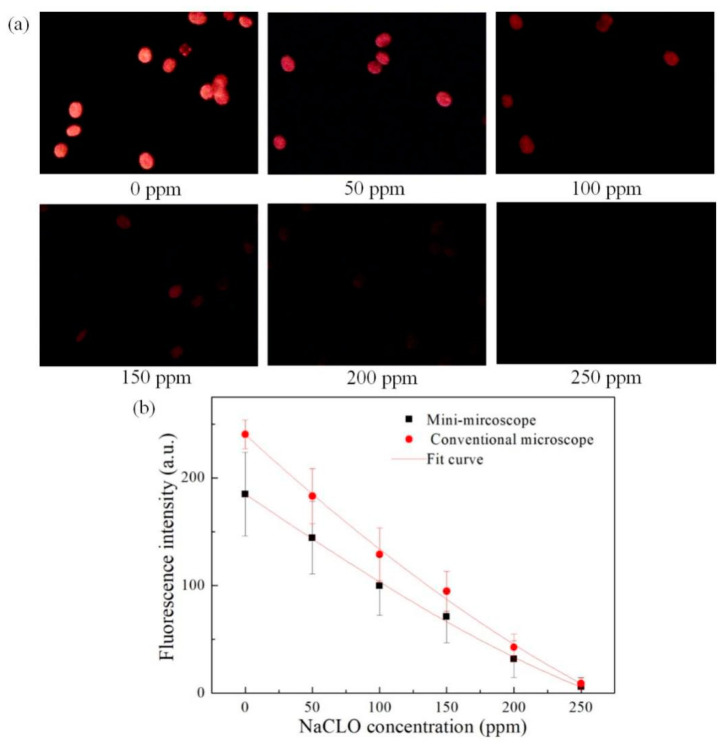
(**a**) Fluorescence image of *Prorocentrum lima* cells treated with 6 different concentrations of NaClO captured by the miniaturized system. (**b**) Comparison of the mean fluorescence intensity calculated by the miniaturized system and the commercial microscope.

Since the intensity of the CF signal is directly proportional to the viability of the cells, the changes of the cell viability were detected here. Six different concentrations of NaClO solution (0, 50, 100, 150, 200, and 250 ppm) were used to inactivate the *Prorocentrum lima* cells (the same samples were placed in six separate tubes, treated for 20 min), and then the fluorescence intensity of the treated microalgae cells was detected by the miniaturized system and commercial microscope respectively. Figure 6a shows the fluorescence images of *Prorocentrum lima* cells treated by six different concentrations of NaClO solution captured by our system. As the figures shown, with the increase of the solution concentration the intensity of the CF signals decreased, which is in line with expectations.

In order to verify the accuracy of the detection results, commercial microscope was used to capture the fluorescence images of the six treated *Prorocentrum lima* samples, and the mean fluorescence intensity of each image was measured by ImageJ software. The mean fluorescence intensity was defined as follows:

Mean fluorescence intensity = the total fluorescence intensity of the region/the area(1)

The calculated mean fluorescence intensity of the above images was shown in Figure 6b (n = 3, n is the number of replicates). From the results, it can be seen that the variation trends of fluorescence intensity detected by this system is consistent with that of the commercial microscope. It is demonstrated that the system has the ability of analyze the changes of the viability of microalgae cells.

### 3.5. Evaluation of Ship Ballast Water Inactivation Effect

Ship ballast water must be strictly inactivated before discharged. However, how to evaluate the inactivation effectiveness of microalgae-laden ballast water efficiently is a major challenge of the ballast water detection. To address it, the following experiments were carried out. The mixture of living and dead *Platymonas* cells (treated by NaClO solution with a concentration of 300 ppm) was used here. Then, the mixed algal solutions (dead and alive cells) with different ratios (dead/total) of 0.2, 0.4, 0.6, 0.8, 1 were prepared, and the BF and CF images under bright-field and fluorescence modes were captured by this system. From the BF images, the concentration of the mixed cell solution can be obtained, and the viability of the cells was obtained from the CF images. Then, the following equation was used to calculate the inactivation rate:

Inactivation rate = (The total number − living number)/total number(2)

**Figure 7:**
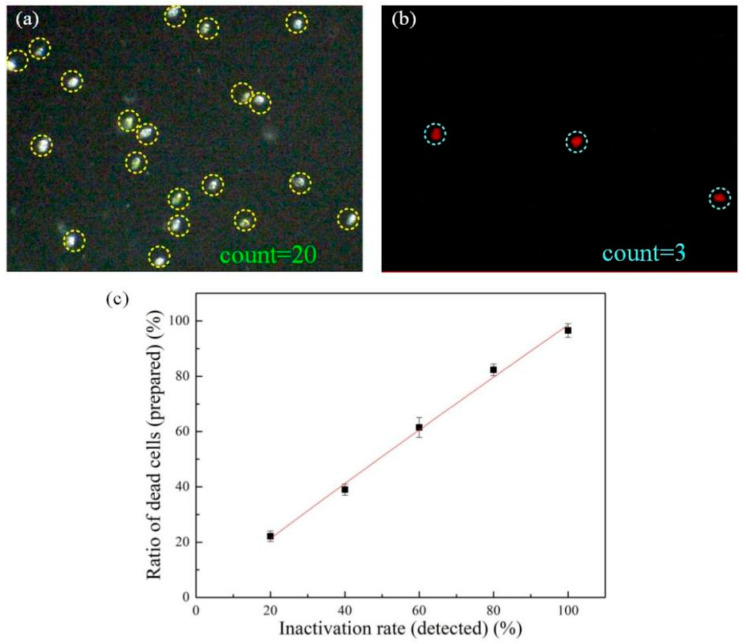
(**a**) BF image of the mixed samples (living and dead *Platymonas* cells) captured by the miniaturized system. (**b**) CF image of the mixed samples captured by this system. (**c**) Comparison of the inactivation rate calculated by this system and the real value.

Figure 7a is a BF image of the mixed samples (living and dead *Platymonas* cells) captured by the miniaturized system under bright-field mode. From the figure, the living cells and dead cells are almost indistinguishable in the bright-field. Using the self-developed counting software, the total number (living and dead) and the concentration of cells can be automatically calculated. Figure 7b is the CF image of the mixed samples captured under fluorescence mode. As mentioned before, the dead cell cannot emit a CF signal, so the cells in Figure 7b are all living cells. Then, the inactivation rate can be calculated according to Equation (2). Figure 7c shows the comparison of the inactivation rate calculated by this system and the real value. It can be seen that the calculated result by our system is close to the real value. The experimental results demonstrate that the proposed method can detect the inactivation effect of ship ballast water rapidly.

### 3.6. Simultaneous Detection of Viability and Concentration

The BF and CF images are often displaced due to cell swimming or lens shake. Therefore, in order to improve the detection accuracy and save time, we fused the BF and CF images to achieve the simultaneous detection of cell viability and concentration. The geometric characteristics and concentration of the microalgae cells were obtained by the BF image, and the viability was obtained by the CF image. Through the registration and fusion of BF and CF images, the concentration and viability of the cell solution can be obtained simultaneously.

**Figure 8:**
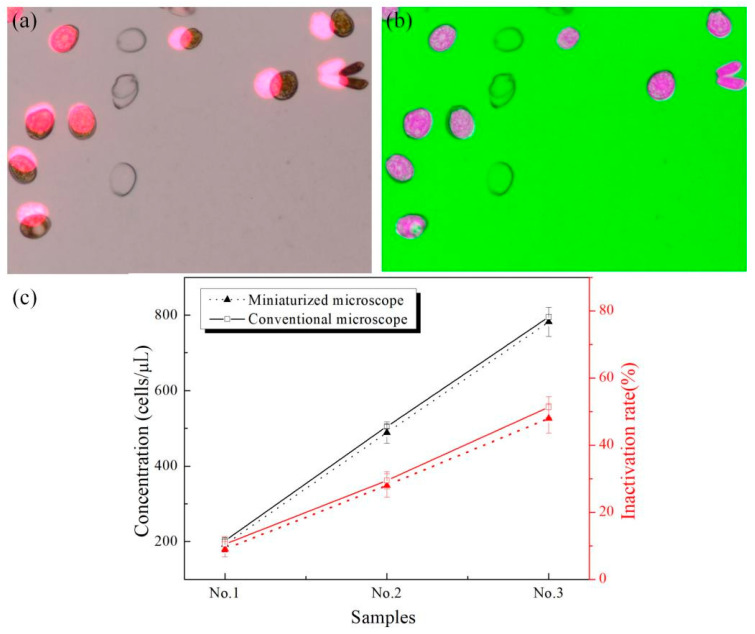
(**a**) Fused image without registration. (**b**) Fused image after registration. (**c**) Comparison with commercial microscope in terms of concentration detection and inactivation rate.

In this paper, firstly, an image registration algorithm based on gray information was used to realize the registration of the two images, and then the weighted fusion algorithm was used to fuse the BF and CF images. Figure 8a shows the fusion results of BF and CF images of *Prorocentrum* *lima* without registration. It can be seen, that there was some displacement between the two images. Figure 8b is the fusion image after registration. It can be seen, that the morphology and viability information of cells can be displayed in the same image, so, the simultaneous detection of the concentration and viability of the sample can be achieved.

In order to evaluate the effectiveness of this method, the following experiments were carried out. Three samples were prepared with the concentrations of 200 cells/µL, 500 cells/µL, and 800 cells/µL respectively. The ratio of dead cells (dead/total) was 0.1, 0.3, and 0.5 respectively. The three samples were detected by our system and the commercial microscope respectively, and the results were shown in Figure 8c. It can be seen that the accuracy of the concentration and the inactivation rates detected by our method is comparable to that of the commercial microscope.

## 4. Conclusions

Based on the principle of chlorophyll fluorescence of microalgae cells, a combination of BF and CF dual images was proposed to achieve the simultaneously detect of the concentration and viability of microalgae cells in ship ballast water. A low-cost miniaturized BF/CF dual imaging microscopy system was designed and constructed using commercial CMOS components. The microscopy resolution of the system is 2.19 μm, the working distance is 2.4 mm, and the magnification is equivalent to the 10× objective lens of commercial microscope. The bottom diameter of the system is 9 cm, the height is 12.5 cm, and the weight is 221.8 g. The system has two imaging modes: bright-field and fluorescence. Under the fluorescence imaging mode, the CF signal of microalgae cells can be clearly obtained, and the viability of microalgae cells can be evaluated according to the intensity of CF signals. Under bright-field mode, BF images of microalgae cells can be obtained and the concentration of the cells can be calculated quickly and automatically. Through the fusion of the BF and CF images, the concentration and viability of microalgae cells in ship ballast water can be rapidly and simultaneously detected. In addition, the inactivation rates of microalgae cells in ship ballast water can also be obtained. Four typical microalgae cells (*Platymonas*, *Chrysophyta*, *Prorocentrum lima*, and *Pyramimonas* sp.) in ballast water were used as the samples for verification. The results show that the miniaturized dual-imaging microscopy system is comparable to commercial microscopy in the detection of the concentration and viability of microalgae cells.

To sum up, the proposed method provides a low-cost, high-efficiency solution for the detection of the microalgae cells in ship ballast water. It should have great significance in ship ballast water management and the marine conservation. In addition, the system can also be extended to the analysis of other microorganisms, and has broad applications such as biomedicine, food safety, and water pollution.

## Figures and Tables

**Table 1:** FWHM of three kinds of microalgae cells (µm).

Microalgae	Conventional Microscope	Miniaturized Microscope
*Chrysophyta*	5.04	4.82
*Prorocentrum lima*	31.33	30.22
*Pyramimonas* sp.	12.86	13.14

## Supplementary Materials

The following are available online at https://www.mdpi.com/article/10.3390/mi12080896/s1.
