# Supplementary material for: Simultaneous Detection of Viability and Concentration of Microalgae Cells Based on Chlorophyll Fluorescence and Bright Field Dual Imaging
Source: Micromachines (Basel). 2021 Jul 29;12(8):896. doi: 10.3390/mi12080896 (PMC8398499; doi:10.3390/mi12080896)
Supplement: Supplementary file 1 [file micromachines-12-00896-s001.zip › micromachines-1290109-supplementary.pdf]

micromachines

MDPI

Supplementary materials

Simultaneous Detection of Viability and Concentration of Microalgae Cells Based on Chlorophyll Fluorescence and Bright Field Dual Imaging

Yanjuan Wang [1,2,3], Junsheng Wang [2,3,*], Tianqi Wang [1,2] and Chengxiao Wang [1]

1 Software Institute, Dalian Jiaotong University, Dalian 116028, China; wangyanjuan@djtu.edu.cn (Y.W.); wtq@dlmu.edu.cn (T.W.); aad1957049044@163.com (C.W.)
2 Center of Microfluidic Optoelectronic Sensing, Dalian Maritime University, Dalian 116026, China
3 College of Information Science and Technology, Dalian Maritime University, Dalian 116026, China
* Correspondence: wangjsh@dlmu.edu.cn

1. System cost list

System cost list

Hardware	Details	RMB(yuan)
LED light source	LZ1-00DB00LED Engin, Inc.<br>651 River Oaks, Parkway San Jose, CA USA	150
CMOS sensor and Camera lens	Sony IMX322	288
Filter(Optional)	EX685/50 M, Chroma ATE Inc., Novi, MI, USA	5000
Shell (3D printing)	High toughness resin 3D Model Technology Co., Ltd<br>Shenzhen China	350

Total: 788 yuan RMB (No filter); 5788 yuan RMB (with filter).

2. Image registration and fusion

In order to achieve simultaneous detection of the concentration and activity of the sample, image registration and fusion are required. In this paper, an image registration algorithm based on grayscale information was adopted. The gray relationship of two images was used for registration, and the weighted fusion algorithm was used for image fusion.

2.1. Image Registration

The BF and CF images are often displaced due to cell swimming or lens shake, therefore, image registration is required. The image registration algorithm based on gray information was adopted here, which includes the following four steps: feature extraction and matching, establishment of spatial transformation model, rapid solution of optimal parameters, and similarity calculation.

2.1.1. Feature Extraction and Matching

Herein, the gray information distribution of images was used as the feature, and the pixel gray intensity was used to quantitatively describe the similarity of images.

2.1.2. Establishment of Spatial Transformation Model

During the photographing process, A coverslip or a microfluidic chip was used to limit the stratification of the cells, so there was basically no displacement of the cells in the Z-axis direction. The displacement was mainly caused by cell swimming and rotation, so affine transformation can be applied in the space transformation model, that is, by translation, rotation, and scaling.

2.1.3. Rapid Solution of Optimal Parameters

The optimal parameter is a set of parameters in the determined spatial transformation model, which can accurately map each part of the image to be registered to the reference image through affine transformation.The methods that commonly used include golden section method, gradient descent method, evolutionary algorithm, etc. The gradient descent method was used here.

For a function f with n variables, the function value after k iterations is

$$f(x_1^k, x_2^k, \dots, x_n^k) \tag{1}$$

Carrying out Taylor expansion on the function of k+1 iteration and ignoring the second and above terms are

$$\begin{aligned} f(x_1^{k+1}, x_2^{k+1}, \cdots, x_n^{k+1}) &= f(x_1^k+\Delta x_1, x_2^k+\Delta x_2, \cdots, x_n^k+\Delta x_n) \\ &\approx f(x_1^k, x_2^k, \cdots, x_n^k) + \frac{\partial f}{\partial x_1^k} * \Delta x_1 + \frac{\partial f}{\partial x_2^k} * \Delta x_2 + \cdots + \frac{\partial f}{\partial x_n^k} * \Delta x_n \end{aligned} \tag{2}$$

In the above equation, $\frac{\partial f}{\partial x_n^k}$ is the gradient vector of function f, which can be expressed by ∇f , and Δx is the change of variable in each iteration, expressed in the form of a vector as follows:

$$\begin{gathered} x_1^{k+1} = x_1^k + \Delta x_1 \\ x_2^{k+1} = x_2^k + \Delta x_2 \\ x_n^{k+1} = x_n^k + \Delta x_n \\ \nabla f = \left[\frac{\partial f}{\partial x_1^k} \quad \frac{\partial f}{\partial x_2^k} \quad \cdots \quad \frac{\partial f}{\partial x_n^k} \right] \\ \Delta x = [\Delta x_1 \quad \Delta x_2 \quad \cdots \quad \Delta x_n] \end{gathered} \tag{3}$$

Substituting formula(3)into(2) to obtain

$$f(x_1^{k+1}, x_2^{k+1}, \cdots, x_n^{k+1}) = f(x_1^k, x_2^k, \cdots, x_n^k) + \nabla f \cdot \Delta x \tag{4}$$

Among them, $\nabla f \cdot \Delta x$ is the dot product of the vector, which can be expressed by the product of the modulus of the vector and the angle of the vector, then the above formula can be expressed as

$$f(x_1^{k+1}, x_2^{k+1}, \cdots, x_n^{k+1}) \approx f(x_1^k, x_2^k, \cdots, x_n^k) + |\nabla f| * |\Delta x| * \cos\theta \tag{5}$$

According to Equation (5), the function value drops the most when $\cos\theta$ is equal to -1, in this case, the gradient vector and the input parameter change in the opposite direction. This is the strategy of the gradient descent method. That is, search in the opposite direction of the gradient change direction. It can be expressed as

$$\begin{gathered} x_1^{k+1} = x_1^k + \Delta x_1 = x_1^k - \alpha * \frac{\partial f}{\partial x_1^k} \\ x_2^{k+1} = x_2^k + \Delta x_2 = x_2^k - \alpha * \frac{\partial f}{\partial x_2^k} \\ \vdots \\ x_n^{k+1} = x_n^k + \Delta x_n = x_n^k - \alpha * \frac{\partial f}{\partial x_n^k} \end{gathered} \tag{6}$$

where α is the step size of each iteration.

2.1.4. Similarity Measure

In order to compare the registration results, we need a standard to measure the registration accuracy of the current parameters. Generally, the similarity is used to judge the similarity of two images. We use the mutual information method of statistical information theory as the basis of Judgment. We believe that the parameter with the largest mutual information value is the optimal parameter, and the search strategy is used to find the result and output it. The following is the derivation process of using image gray information to calculate the mutual information value between images, and to measure the similarity.

Assuming that there are n random phenomena in a given case, and their probabilities of their occurrence are p_1、p_2、$\cdots p_n$, respectively, then the Shannon entropy is defined as

$$H=\sum_i p_i \log_2 \frac{1}{p_i}=-\sum_i p_i \log_2 p_i \quad (7)$$

The above Shannon entropy can be used to calculate the amount of information contained in an image. The probability of occurrence in the equation corresponds to the distribution of gray values in the image. The probability distribution of gray values is represented by the number of occurrences of a certain gray value in an image divided by the number of occurrences of all gray values. The Shannon entropy can also be used to express mutual information, as shown in Equation (8). The mutual information of images A and B can be expressed by the Shannon entropy

$$I(A,B) = H(A)+H(B)-H(A,B) \quad (8)$$

In the Equation (8), $H(A)$ represents the entropy of sample A, $H(B)$ represents the entropy of sample B, and $H(A,B)$ represents the joint entropy of the two. According to equation (7), the above entropies can be expressed as

$$H(A)=-\sum_a p_A(a)\log_2 p_A(a) \quad (9)$$

$$H(B)=-\sum_b p_B(b)\log_2 p_B(b) \quad (10)$$

$$H(A,B)=-\sum_{a,b} p_{AB}(a,b)\log_2 p_{AB}(a,b) \quad (11)$$

Among them, $a \in A$, $b \in B$, $p_A(a)$, and $p_B(b)$ represent the independent probability distribution of samples A and B. $p_{AB}(a,b)$ is the joint probability distribution of A and B.

Substituting the above Equations(9)–(13) into Equation (8) is

$$I(A,B)=-\sum_a p_A(a)\log_2 P_A(a)-\sum_b p_B(b)\log_2 P_B(b) +\sum_{a,b} p_{AB}(a,b)\log_2 p_{AB}(a,b) \quad (12)$$

In the above equation, $p_A(a)$and $P_B(b)$ can be represented by the normalized histogram of image A and image B, respectively. $p_{AB}(a,b)$ can be represented by a normalized joint histogram of images A and B. The normalized histogram of an image is

$$p(r_k)=\frac{h(r_k)}{n}=\frac{n_k}{n} \tag{13}$$

where n_k is the number of occurrences of a certain gray level r_k, and n is the total number of occurrences of all gray values. The normalized joint histogram of the two images A and B is obtained by the equation

$$p_{AB}[a(i,j),b(i,j)]=\frac{h_{ab}[a(i,j),b(i,j)]}{\sum_{i,j} h_{ab}[a(i,j),b(i,j)]} \tag{14}$$

where $h_{ab}[a(i,j),b(i,j)]$ is the joint histogram function of images A and B. $a(i,j)$ and $b(i,j)$ respectively represent a pair of gray values of two images in the same position. $h_{ab}[a(i,j),b(i,j)]$ represents the occurrence of the same gray level value pair. Therefore, the normalized joint histogram of two images is to calculate the occurrence of the same gray level in the same position. By substituting the above Equations (13) and (14) into Equation (12), the mutual information value can be obtained according to the gray level information of the input image.

2.2. Image fusion

The fused image can get a more accurate and comprehensive description of the target, providing a more intuitive image. This is beneficial for detection. The commonly used image fusion algorithms include weighted fusion, wavelet fusion, pyramid transform fusion, and so on. Herein, the weighted fusion algorithm was used. The images obtained by different types of sensors are also different. According to the actual situation, we assign weights of the images. Assign greater weight to the parts that are rich in details or need to be highlighted.

We assigned different weighting coefficients according to cell type and fluorescence intensity. In the CF image, because the fluorescent spot was mainly used as a reminder of cell activity, and considering its black background, we assigned a smaller weight to it. The BF image contains detailed information such as the color, shape, and structure of the algae cells, so we assign a larger weight value to it.

3. The accuracy of the cell concentration detection of this system

In order to evaluate the accuracy of the system's automatic concentration detection algorithm, the following experiments were carried out. Five different concentrations of Platymonas samples were prepared, which were 100 cells/ μL, 300 cells/ μL, 500 cells/ μL, 700 cells/ μL, and 900 cells/ μL. The concentration was detected by microscope and the system respectively, and the samples of each concentration were detected three times. The detection errors of the system were calculated based on the results of the microscope, and the calculated results are as follows:

**Samples**	**100 cells/μL**			**300 cells/μL**			**500 cells/μL**			700 cells/μL			900 cells/μL		
**Method**	**System**	**Microscope**	**Error**	**System**	**Microscope**	**Error**	**System**	**Microscope**	**Error**	**System**	**Microscope**	**Error**	**System**	**Microscope**	**Error**
1th	108	102	0.0588	285	297	0.0404	524	507	0.0335	672	689	0.0247	925	912	0.0143
2th	105	103	0.0194	279	291	0.0412	485	508	0.0453	653	691	0.0550	953	921	0.0347
3th	103	105	0.0190	303	294	0.0306	489	513	0.0468	687	712	0.0351	938	909	0.0319
Mean Error: 0.0354															

Therefore, compared with the microscope detection method, the detection error of this system is 3.5%.
